# Supplementary material for: Transcriptome Analysis and miRNA Target Profiling at Various Stages of Root-Knot Nematode Meloidogyne incognita Development for Identification of Potential Regulatory Networks
Source: Int J Mol Sci. 2021 Jul 12;22(14):7442. doi: 10.3390/ijms22147442 (PMC8307930; doi:10.3390/ijms22147442)
Supplement: Supplementary file 1 [file ijms-22-07442-s001.zip › ijms-1270631-supplementary.pdf]

Supplementary data

Table S1. Overall statistics of mRNA gene expression (FPKM ≥ 0.3).

Type	Number	%
**Total Transcripts (Gene Model)**	20,365 (19,212)	100.00
**Mapped genes**	18,637	91.51
**Expressed genes**	17,423	85.55
**Non mapped genes**	1,728	8.49

Figure S1. Enzymes coded by mRNA sequences of *M. incognita*. A. Our study B. Reference sequence PRJEA28837.

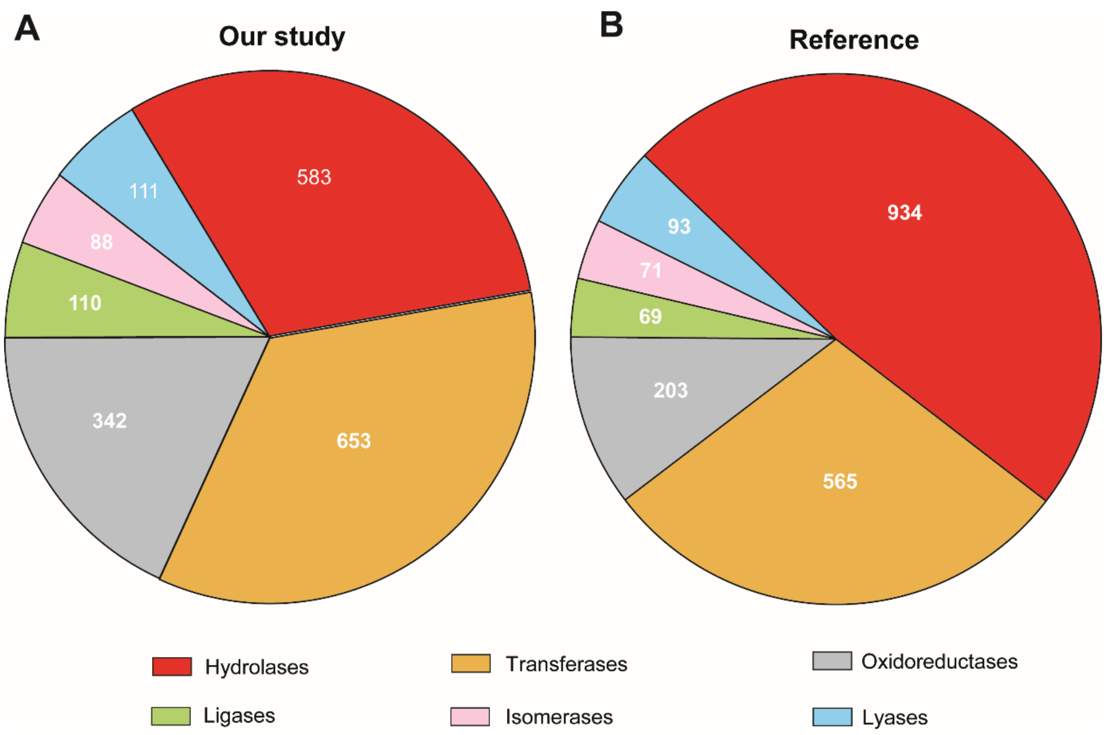

Table S2. Interproscan statistics comparison between mRNA contigs in the present study and available database.

	Number of sequences				
Type	Novel miRNA targets	Known miRNA targets	Common	3program targets	Reference
without IPS	51	74	431	6771	6315
with IPS	195	321	1676	0	14050
with GOs	112	170	893	0	7919

Figure S2. Stage-wise analysis of gene expression; Common; Egg; J2; J3; J4; and Female.

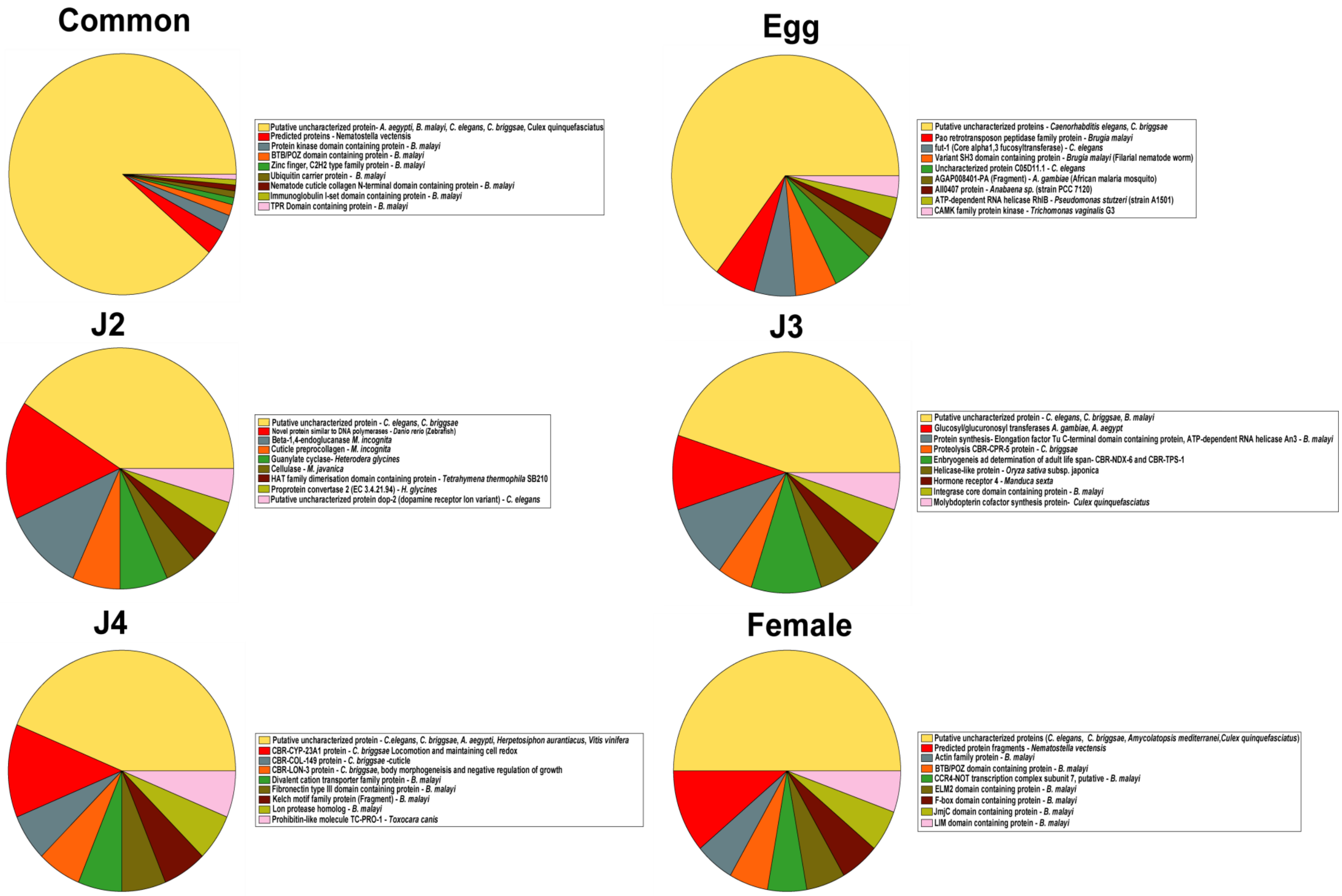

Figure S3. Heat map indicating expression of mRNA at all stages indicating expression (FPKM). Hierarchical cluster analysis provided with the MeV software indicated J3 and J4 stages to have similar mRNA profiles which were related to Egg, J2 and female in the descending order of similarity. Box and arrow indicates highly expressed mRNA transcripts id (Correspond to Table 4 hit description) from developmental stages of *M. incongita*.

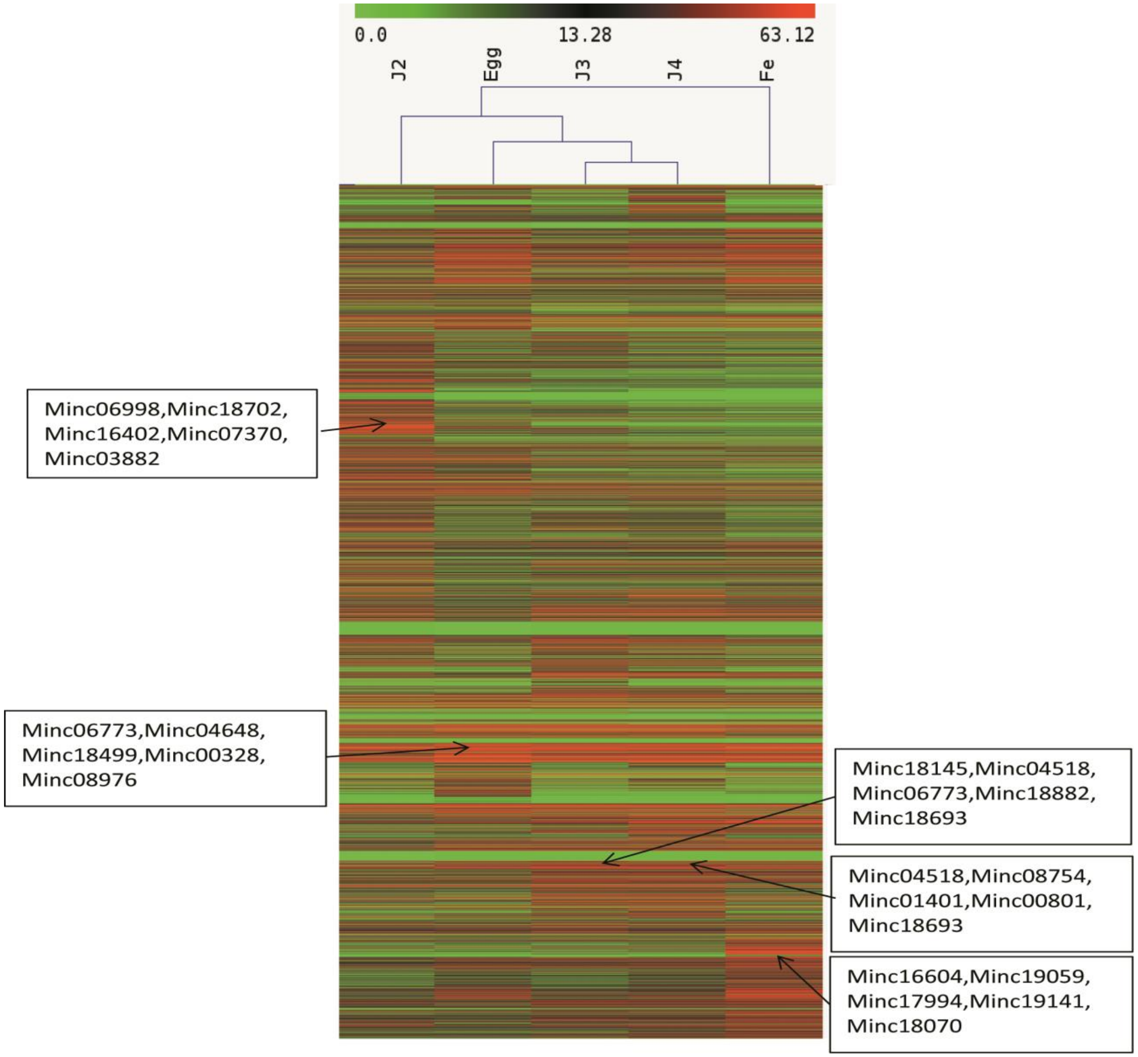

Table S3. The 3’UTR summary of *M. incognita*.

Type	Total	N (bp)	Min (bp)	Max (bp)	Mean (bp)
3' UTR	20,365	1,059,617	0	5,000	428.2
3' UTR (length ≤ 10)	1,730	83	0	10	0
3' UTR (N< 1% and length ≥ 10)	17,177	74	11	5,000	393.6

Figure S4. Real-time expression of commonly expressed mRNA. Egg, J2, J3, J4 and Female Stages.

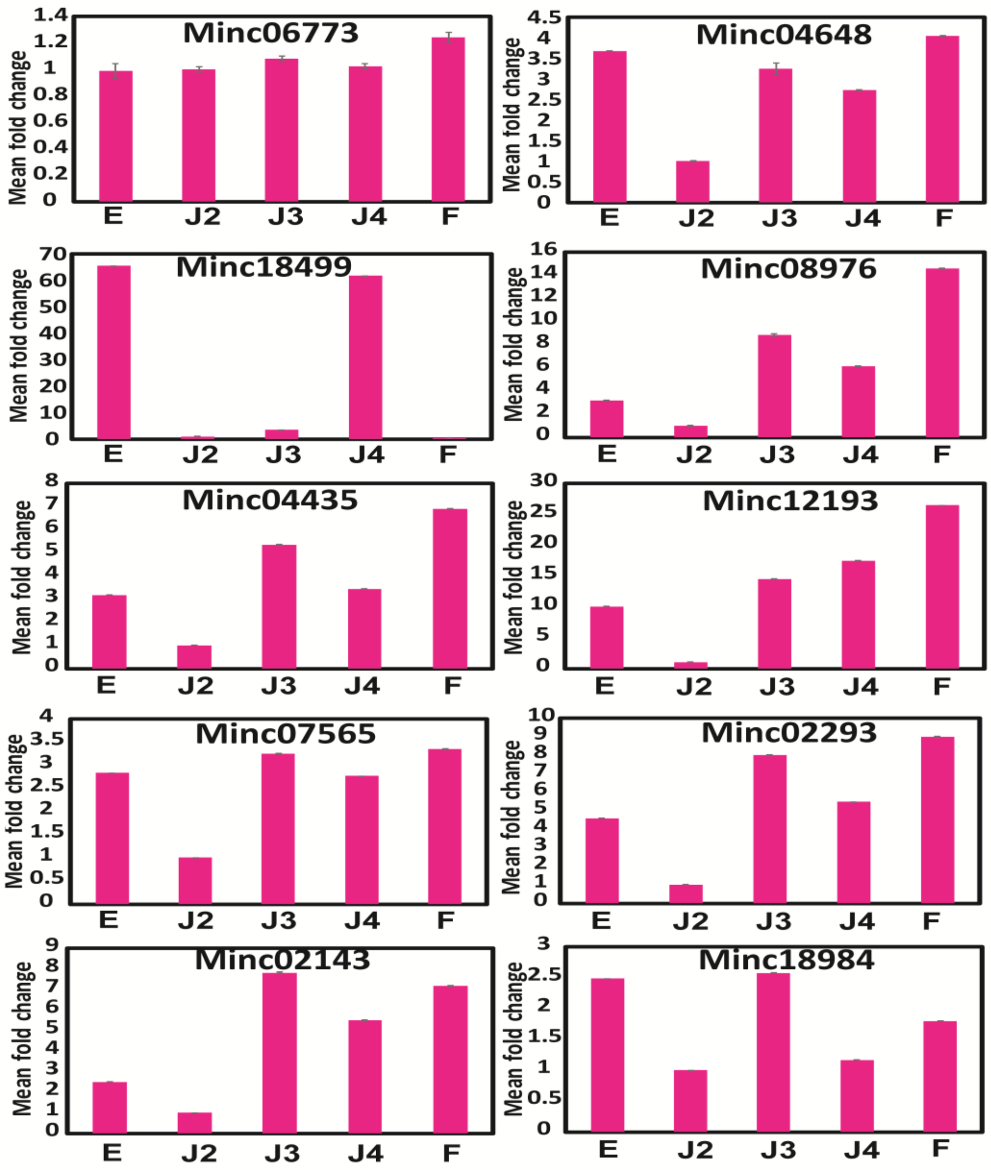

Table S4. List of miRNA targeting mRNA primers used for qPCR analysis.

Stage	MiRNA-mRNA	Forward	Reverse
Egg	Minc13648	CATTCCGACGCTTCCCTAAA	AACATTCGGTGCAGATTCAAAC
	Minc08819	CCTCCACATTGGCTTGAACTA	ACGACCAACTAAAGGATGAGAC
	Minc00198	AGGTCCTTTGTGGAGACAATC	CCGCCACACACCTTCTTTA
	Minc03013	ATGCCGTCCGTGTCATAAA	TCCAAGTTCATCCCTCAATCC
	Minc18132	TGGGAAGAAGGAAGTGAAGAAA	TCTTGTTGGACTCTCCAAATCT
	Minc17860	CCGATTCATCGTCCTCCAATAC	CCGTCAACCAAGAACGAAGA
J2	Minc06090	CTAGGGTGTGCTAGAGCATATTG	TTGCCAGGAGGATTGTATTGG
	Minc01401	TGGATAGGGAATTGACGATGTG	CTGTACGATTCCCACCACTAAC
	Minc09926	GTGTTGGAGAAGTATGGGTCTT	GATTTGATTTCCAGCCGATGAAT
	Minc00014	TGGTGAAAGCAGAGATGAAGAG	ATTCGCCTGGAGCACAATAG
	Minc10465	GGGTGAAGAATTAGAGAATAAGTGGA	CTTGTCCTGATTCGCGATCTAA
	Minc13117	CCAAAGGTTCTGTTGCTTTCC	GATCACAAGCAGCCATCATATTC
J3	Minc00091	CACTGATCTCGGAATGCTTCA	GCGTCACCTTTGATGGTTTG
	Minc00288	CGCTATAATGACCCGCTTGA	ATGACCAACATCATCCTGTCC
	Minc03384	TGGTGGACAGTGCAAGTATG	GTGTTGCAGGCAAATGAGAAG
	Minc13301	ACAAACGGGCCAACAGAA	ATGACCAAGACGGCACATATAG
	Minc10047	GTGCTGCTCTTCAAACAACTG	TTCCTCACGACGTTCTCTAGTA
	Minc18726	AAGGACGACGACAAGAAGAAG	TCCAGCCTTCGATGTCTTTC
J4	Minc16259	GATGGAATTACCAGGGACTCTTAC	GACATATTCCTTCCCGTCATCTT
	Minc01292	GCTGGATGCGAAAGAAATACAA	GGGAATGAGTAAGAAGCAGAGG
	Minc02324	GGTCGTGACATGGATCTTACC	GCCCAATCGCCACAATTTC
	Minc02683	TCGGTTGGGAAGGCTTATTG	GAAGGAAACAGTGCGCAAATC

	Minc00193	GGTACAAACGAAGGAGCATCA	ACGTTCTCTTTGGACCCAATC
	Minc00661	TAAGGAGCTTGTCGACTTTGG	CGAATTTGGAGGCCCAGTAA
Female	Minc12284	AGGGCCGAGAGGAGATAAA	ATCGCCAATATCACCAGGAAG
	Minc01496	TGTTGTCCTTGTATGAGGGTTAG	TGCCTTCATCGGCTTCTTT
	Minc04950	GCGCAGATCTAGGTGTTTACT	GCAGCCGGGTTCTTTACATA
	Minc03829	AGCCAGCCGCGATTATATG	CAGCAGTCCAAGAAGAGAAAGA
	Minc11857	GCCGTTGAGGACGAAACTAA	CCTCAGCATCAGCAGGTTT

Table S5. List of highly expressed mRNA primers used for qPCR analysis.

Stage	mRNA	Forward	Reverse
Egg	Minc11396	GCAAACTATATCTACTGAAATGTCCAAG	AAAGTGCCAGATGGTGGTG
	Minc03738	ACAAAGATTCTTGCCAAATGTC	CTTGCATCTCCTTGGAATGAT
	Minc10990	GAAAGCAGATTGGTAAGGCAATATC	AATGGCCTAGCCTCCAAATC
J2	Minc01592	GCACAGTCGAATTGTAAAGATACTC	CCAATCCAATACAATCACAAGCA
	Minc19090	GAATGAGCCTGGTTGTGATTG	TGTAAGAGGGCATCCAAGAATAG
	Minc05278	ACAACAACCGAGCCATCTAC	GGTGGTTGAGCGCAATTAAAG
	Minc01593	CATGCCCTTCCATGTTCAATC	GGATCTTGTGTCTCTCGCTTAG
	Minc09446	GCTGCAATGGGTGTAGAGTAT	AACTCTATCGCGGCCTTAAC
	Minc18141	ACTGTTGAGGGAGTTGAAGAC	CCTTCCAAGGTCCCAAAGAA
J3	Minc16891	TCCTCGAGTAAAGGCAAAGAA	GCAGCTCTGCACATCTACTA
	Minc00205	GAAGCTCCACAGGCAATCT	AGGCTGATCCTCCCATTTATTC
	Minc15344	TTCTGCCTCTTATGACGAACC	GGGCAACCAGGACAATGA
J4	Minc06871	CTGAACGACGTGCTAGTCATAG	GGGCTGAATCCAACCATACT
Female	Minc08697	GGTGGAAATGACGAGGAATTTG	GACAATCAATGTAGGCACAACC
	Minc18984	TTGATTCTAAAGCCAGGGAACT	CCATGTAATGACCGTCTCTATCC
	Minc02270	TATATTCTGCAATTTGTTGTAACGA	ATCTTGAGCAACATGGGGTTG
	Minc18856	GATCCACTTTGCCATGTTTGTC	GCGTGTGTTGCTAGACATTTG
	Minc07322	CAAATACTTGTGGAGGGCTTTG	CTCAACAGGTCCGACAACAT
	Minc02526	AATGTGATGAACCCGAGGAC	GCTACGCTTCCGCTTCTTA
	Minc12762	GCCCTAATGCGGAATATGTTATG	CTAGGCCAGTTCCAGTTTCTT

Table S6. List of commonly expressed mRNA primers used for qPCR analysis.

Stage	mRNA	Forward	Reverse
Common(Egg, J2, J3, J4 & Female)	Minc06773	TCCTCACTGAACGTGGTTATTC	GCTCAAAGTCCAAAGCAACATAG
	Minc04648	AAGGATCCTAACATGCCGAAG	CTTCTTGATGCGCTCTCTGT
	Minc18499	CTCGTGAACCCTGTATCATCTG	GGAGTTCCTTTATCTCCGTTCTT
	Minc08976	AGCTGGTGTTGAAGTCGAAG	CACCTCCACCAGATCCAATTC
	Minc04435	AATCCGAACTTCTGTCCACTTT	GTCGAGTTTGTTGCGTTTGG
	Minc12193	GTCTTGTGGCGTTGTGACTA	CCCTGTTGGTCCTGAAACTC
	Minc07565	GTTGTCGCTACTGGTGATGA	ACCGAGACGAATTGGATGAG
	Minc02293	CAAGCGCAAGCTGGCTAAG	TTTGTGCGGCGCCAATG
	Minc02143	GGCGGTGCTTATGGAAAGA	TGCTCCTCATGGTGTGATTG
	Minc03634	CGCTCAATCCGCTTACTTCA	GAAATTTAAAGCGAACCTCATCCC

Table S7. List of selected and validated miRNA targeting mRNA; microRNA sequence,mRNAid, conting region and fuction.

Stage	MicroRNA	MicroRNA sequence(5'-3')	mRNA	Site region (V1)	Function
Egg	MIN00371	TTTGAAACTGAAGCAAATTTGC	Minc13648	MiV1ctg706:8858..17325	UDP-glucuronosyl/UDP-glucosyltransferase
	MIN00067	AGCCCAATGTACCGTCGAGCTAT	Minc08819	MiV1ctg297:29557..30585	Patched family protein (*Brugia malayi*)
	MI03228	TGGAGCAGTTGGGAGCTATGAG	Minc00198	MiV1ctg2:168090..170136	Beta-1,4-N-acetylgalactosaminyltransferase bre-4 (*Toxocara canis*)
	MI02542	TACATACACACACAAATAT	Minc03013	MiV1ctg59:90607..98430	*Schistosoma mansoni* tyrosine kinase partial mRNA
	MI00171	AACGAAGACCGCGGCGGAGCTGT	Minc18132	MiV1ctg1741:3017..5273	Beta-1,4-galactosyltransferase
	MI02288	GTGAATGGATAAAGCGATGTTAC	Minc17860	MiV1ctg1597:3754-5934	Hypothetical protein DICVIV_00975 (*Dictyocaulus viviparus*)
J2	MI01348	CATGGGGAATGGGATGGGGCAG	Minc06090	MiV1ctg163:15984..16437	VAP1 protein (*Globodera rostochiensis*)
	MI03464	TGTCCTTTGGAGGTCGCTCTTTCA	Minc01401	MiV1ctg21:214723..219350	Nematode cuticle collagen, N-terminal
	MI01348	CATGGGGAATGGGATGGGGCAG	Minc09926	MiV1ctg361:11891-12686	Unknown function
	MI02028	GGAGAAGACGGGGGTGGTGGTGGG	Minc00114	MiV1ctg1:62506..67067	Low-density lipoprotein (LDL) receptor class A, conserved site
	MIN00235	GTTCTTGATCGTTGACGCAACCAC	Minc10465	MiV1ctg398:31698..33820	F-box domain
	MIN00380	TTTTCCCCTTTTTTCTCTCTTTG	Minc13117	MiV1ctg640:21081..22810	*H.contortus* beta tubulin

**J3**	MI02061	GGAGGGGTGCAGGCGCGG	Minc00091	MiV1ctg0:3627 10..367915	alpha-(1,6)-fucosyltransferase-like *Ciona intestinalis*
	MI02049	GGAGGAGGAGGAGGATTGGGTGG	Minc00288	MiV1ctg3:2322 23..232535	hypothetical protein CRE_13056 *Caenorhabditis remanei*
	MI00880	AGGGGGAGGTGGTAGTGGATGT	Minc03384	MiV1ctg69:158 477..164172	unnamed protein product (*Chondrus crispus*)
	MI02049	GGAGGAGGAGGAGGATTGGGTGG	MInc13301	MiV1ctg662:36 947..37809	Cytochrome c1, transmembrane anchor, C-terminal
	MI02411	GTTGGTGGAGGAAGTGGAGGTGG	Minc10047	MiV1ctg368:42 641-44025	P. Ribosome recycling factor family protein - *Brugia malayi* (Filarial nematode worm)
	MI00880	AGGGGGAGGTGGTAGTGGATGT	Minc18726	MiV1ctg2241:2 372..3507	translation initiation factor IF-2 (*Corynebacterium matruchotii*)
**J4**	MI03255	TGGAGGTGCAGGGCGAGGTTTTC	Minc16259	MiV1ctg1130:1 3616..14117	Chromosome transmission fidelity protein 8
	MI03615	TTGAAGAGGATTGAAGGGGAGGG	Minc01292	MiV1ctg19:204 039-205420	P. Nk homeobox protein - Culex quinquefasciatus (Southern house mosquito)
	MI01808	GAGGGAGAAGGGGATGTTGAC	Minc02324	MiV1ctg42:161 023..161238	Unknown function
	MI03255	TGGAGGTGCAGGGCGAGGTTTTC	Minc02683	MiV1ctg52:502 70..57837	Band 7/stomatin-like
	MI02241	GTAGAATTGTTGGTGGATGGCTT	Minc00193	MiV1ctg2:1446 21..146119	hypothetical protein H257_06861 (*Aphanomyces astaci*)
	MI03255	TGGAGGTGCAGGGCGAGGTTTTC	Minc00661	MiV1ctg9:4080 6..42545	Ubiquitin-conjugating enzyme E2 2 (*Caenorhabditis elegans*)

**Female**	MI01064	ATGAAGAATGGTATGGGCAAAA	Minc12284	MiV1ctg553:11588..13235	*Brugia malayi* Nematode cuticle collagen
	MI01396	CCTCAACAGCAGCAAACTCA	Minc01496	MiV1ctg24:46974..53500	Sterol-sensing domain
	MI00304	AAGGGGGAAGGGACGGGTGGCA	Minc04950	MiV1ctg119:75700..76731	Profilin-1 (*Caenorhabditis elegans*)
	MI02634	TAGGGGGTGGAGGTGGGG	Minc03829	MiV1ctg83:61065..63639	Hypothetical protein CBG01887 (*Caenorhabditis briggsae*)
	MI02712	TATGGCTTCGATTCCTGATG	Minc11857	MiV1ctg515:35495..37196	*Meloidogyne javanica* putative secretion protein MJD15 mRNA
